# NoFumo+: Mobile Health App to Quit Smoking Using Cognitive-Behavioral Therapy

**DOI:** 10.1155/2024/8836672

**Published:** 2024-09-26

**Authors:** Patricia García-Pazo, Joana Fornés-Vives, Albert Sesé Abad

**Affiliations:** ^1^ Department of Nursing and Physiotherapy University of the Balearic Islands (UBI), Ctra. Valldemossa, km 7.5, Palma E-07122, Spain; ^2^ Health Research Institute of the Balearic Islands (IdISBa), Ctra. Valldemossa, 79, Palma E-07120, Spain; ^3^ Department of Psychology University of the Balearic Islands (UBI), Ctra. Valldemossa, km 7.5, Palma E-07122, Spain

## Abstract

This paper describes the development and test of a smartphone application to quit smoking using cognitive behavioral therapy (CBT). The tool includes recommendations from US Clinical Practice Guidelines (USCPG), drawing on the potential of smartphones and complying with the health App (mHealth) assessment standards. The mHealth created, called NoFumo+, is structured by 4 weeks treatment, implements the USCPG 5A recommendations (ask, advise, assess, assist, and arrange) and incorporates a CBT. It also includes complementary information, monitoring of the smoking behavior, social support for users, proposals for alternative activities to smoking, and innovative gamification to encourage and reward adherence. To technical development, a multidisciplinary team was formed (healthcare, research, and software engineers) that made theoretical decisions on both technical issues and the incorporation of therapeutic techniques. The validation was carried out in two phases; the first in the laboratory by a group of experts in information and communication technologies and CBTs (*n* = 15) and the second, a field study with smokers (*n* = 10). The standards for the development of mHealth recommended by the Andalusian Healthcare Quality Agency and the App quality evaluation guidelines of the Catalonian ICT Foundation for Social Health were used as assessment protocols by the experts' panel and the smokers' group, respectively. Experts' assessment results were satisfactory and some improving changes were suggested, such as to add more gamification elements. The group of smokers rated the mHealth as 100% easy to use and effective for quit smoking and understandable by the 83.3%. They also found No Fumo + quite useful to have the information available at all times. The obtained evidence after a complete two-phased validation study, with experts and potential users, shows a mHealth with high quality and easy to use. Finally, investigation project registered in ClinicalTrials.gov with reference to this trial is registered with NCT045402004.

## 1. Introduction

Tobacco use is one of the most serious health problems worldwide and the main cause of various respiratory, cardiovascular, and oncological diseases [1]. In Spain (2021), the prevalence of daily tobacco consumption is 32%, [2] and the Balearic Islands has the seventh highest rate of smoking prevalence in Spain [3]. Smoking produces a high morbidity and mortality rate, given that about half of all tobacco users die from it [4]. Tobacco is not only harmful to smokers but also to the people around them, many of them children. In addition to being a serious health problem, smoking places a heavy economic burden on people around the world. In Europe, by the year 2027, estimations indicate that direct costs (hospitalization, inpatient rehabilitation, outpatient care, and medication) will reach 36 billion euros, whereas indirect costs (death, loss of work days, and early retirement) will reach 43 billion euros [5].

Smoking is an addiction that is usually extremely difficult to break, and it has a high relapse rate, especially among patients with chronic diseases [6]. Due to the magnitude of this problem, healthcare personnel consider it a priority to treat smoking addiction in order to improve smokers' health [7]. For this reason, in our context, the Spanish Society of Pneumology and Thoracic Surgery (SEPAR), in accordance with the Clinical Practice Guidelines of Fiore et al. (CPG) [8], states that healthcare professionals, regardless of their discipline (doctor, nurse, and psychologist, among others), should begin to treat tobacco use when the smoker visits any healthcare service [9]. In Spain, between 15 and 27% of patients admitted to hospital are smokers [10]. In this regard, the hospitalization period of smokers is considered a “teachable moment” to modify the smoking habit [11–13]. There are two basic reasons for taking advantage of this period. On the one hand, patients may be more motivated to remain abstinent when their health is threatened [12]; on the other hand, smoking is prohibited in hospitals (in Spain, Law 42/2010 of December 30th, which modifies Law 28/2005 of December 26th) [14].

Moreover, in this situation, smokers are surrounded by healthcare professionals who can advise them and promote interventions to overcome their addiction [15]. These interventions could be carried out through effective actions, such as brief advice with the mnemonic rule of the 5A's recommended by the CPG: ask, advise, assess, assist, and arrange; that is, ask about consumption, advise the person to quit, assess and help to elaborate a specific action plan, and arrange follow-ups [8, 16].

However, it usually is not implemented in many hospitals; specifically, a study conducted in 13 Spanish hospitals indicates that only 4.6% of hospitalized smokers received the full brief advice [17]. There are several reasons why brief advice is not implemented in hospitals: professionals responsible for its application often lack the necessary skills, motivation, or trust in these treatments, and hospital management typically does not facilitate this type of intervention [18–21]. The evidence-based treatments that have shown the greatest efficacy in quitting smoking include pharmacological therapies (varenicline, bupropion, or nicotine replacement therapy), cognitive behavioral therapy (CBT), either group or individual, or the combination of the two [22].

Generally speaking, CBT to quit smoking is offered in multicomponent programs designed not only to stop smoking, but also to maintain abstinence [23]. These programs highlight components such as motivational techniques, behavior analysis, stimulus control, response prevention, social support, problem solving, social skills training, and stress management techniques. CBT also includes cognitive techniques such as cognitive restructuring and thought stopping [23, 24]. According to a review carried out by Almaraz and Alonso [25], the techniques that have shown greater effectiveness in quitting smoking are analysis of the smoking behavior by identifying triggers, social support to maintain motivation, and stress management techniques to prevent relapse. In accordance with a recent systematic review with a meta-analysis, cessation rates are strongly predicted by the number of behavior change techniques used [26].

In the context of this study, the different areas of public health in the Balearic Islands (Spain) have been applying a standardized multicomponent CBT program to quit smoking that incorporates the aforementioned interventions in a face-to-face format, either individually or in groups [27]. That program is an adaptation of the one proposed by Becoña [23, 24], which has shown good results in different studies obtaining abstinence rates of 30% and 41% at 12 months of follow-up [28, 29]. As in other therapy of substance use disorders, these types of multicomponent programs foster the practice of healthy behaviors such as physical exercise and adopting a healthy diet [30, 31]. In general, these programs have mainly been developed for outpatient settings [32, 33] although they have also shown their efficacy in the hospital context [7, 34]. However, the face-to-face application of CBT also has some disadvantages, such as high cost, rigid norms (the user must adapt to the timetable of the sessions), the limited duration of the treatment, poor accessibility, not availability at any time, lack of viability in relapse situations, putting extra pressure on smokers, or accessible way [35–37].

Therefore, it would be desirable to find other alternatives overcome these difficulties, without reducing the effectiveness of the treatment. In this context, digital interventions are experiencing significant growth due to their capacity to address health-related issues, utilizing the capabilities of mobile devices, including smartphones, as well as other wireless tools such as patient monitoring devices and digital assistants. All of these digital methods are commonly named as mobile health (mHealth). These devices, through their applications, known as Apps, can provide meaningful support in the treatment of health problems [38, 39]. Smartphones are currently one of the most widely used electronic device by the general population. In Spain, around 93% of the adult population has one [40].

The main advantage of these smartphone applications is their ability to reach a large population by reducing location and cost barriers [41]. In addition, treatments can be adapted to users, providing them with rapid assistance, anonymity (e.g., benefit in pregnant smokers), offering constant advice and access to therapies at any time, thus increasing the possibility of modifying unhealthy behavior from a distance [42–44]. For this therapeutic purpose, smartphones make it possible to implement complex functions, including audio and video, two-way communication, and additional content when an Internet connection is available [45, 46]. These devices also provide the option of using other online applications, such as a chat to provide social support to users through a forum [47, 48] or a pedometer to provide feedback and encourage physical activity [49, 50]. An international taxonomy with demonstrated efficacy [51] has been described and recommended to adapt behavior change techniques to quit smoking to a smartphone App format [52].

The main weakness of the apps is that 77% stop using them within 72 hours of downloading them, which explains the low adherence to treatment [53]. To counteract this problem, they use notifications as a reminder or warning [54], as well as gaming elements (“gamification”) such as leaderboards, badges, rewards, and avatars to increase patients' encouragement and hence improve effectiveness [55–58]. The motivating and reinforcing capacity of gaming activities has been proven in the literature [59] since when applied within the framework of behavior modification [60], they can have a positive impact on health promotion and maintenance as, for example, to reduce alcohol consumption or increase physical activity [61].

Currently, there are many unresolved questions due to the lack of further research and the heterogeneity among such devices that hinders the study. However, the common consensus of all digital tools lies in developing a user-centered tool and defining the commitment required from the user to achieve the objective for which it has been developed. In the case of an mHealth that applies therapeutic principles such as CBT to pursue behavioral and cognitive change, user's commitment is considered similar to the face-to-face format, which is completing the treatment [38].

In Spain, when developing and implementing an mHealth, organizations such as the Andalusian Healthcare Quality Agency [62] and the Catalonian ICT Foundation for Social Health [63] make recommendations about the design (icons, images, text, videos, etc.), usability (ease of use), and security of the application before putting it into operation. In addition, after evaluating the application and confirming that it meets a series of requirements, identifies it with a quality seal [64].

In the field of tobacco addiction, the use of an mHealth as a therapeutic resource is widespread, both in terms of the number of apps on the market, over 400 for Android or iPhone operating systems [64], and the number of downloads, nearly 800,000 in one month in English alone [65]. However, even taking into account the potential of these devices for implementing these therapies, applications to quit smoking have exhibited significant scientific and therapeutic shortcomings because, in general, they do not adapt to the basic 5A's recommended by the CPG [8]. For example, they do not give brief advice about drop out or guidance about pharmacological treatments [66, 67]. Furthermore, most mHealth do not implement CBT [68, 69]. The ones that do are not free, and not all of them include an analysis of the smoking behavior [70]. Moreover, they do not include social support in a generalized way; only between 2.7% and 17% provide this support [66, 71, 72], and only 4% use gamification [68].

In light of the shortcomings found in the literature on the quality of smartphone mHealths to quit smoking and committed to the development and scientific advancement of digital devices in health. The study is aimed to provide a detailed account of the steps taken in the “NoFumo+” mHealth's creation, including the components it encompasses, its functionality, and the usability and satisfaction tests conducted with groups of experts and potential users prior to its pilot implementation.

## 2. Methods

This study was approved by the Ethics Committee of the Balearic Islands (reference no. IB 3865/19). The consent was obtained written.

The mHealth development to quit smoking started from the choice and theoretical justification and the CBT program selection and its operationalization. After establishing the therapeutic approach, we proceeded with the technical development of the application, followed by its technical and theoretical validation through expert judgment (Phase 1). Finally, a field study was carried out with smokers to obtain evidence of an adequate understanding, usability of the mHealth functioning, and satisfaction (Phase 2). The application was developed throughout 2018-2019, with the first version available for the Android operating system in October 2019 and for Apple devices one month later.

### 2.1. App's Theoretical Foundations and Design

The theoretical framework in which the mHealth was developed is based on the treatment recommended by the CPGs proposed by Fiore et al. [8] and its adaptation to the hospitalized smoker by SEPAR [9]. The techniques implemented followed the 5A's of the CPG (ask, advise, assess, assist, and arrange), and Becoña's multicomponent CBT program [24]. Two existing online applications were added to this program, namely, “Google Fit” as a pedometer, and “Google Chat” to provide social support to users. On one hand, tracking physical activity is associated with an increase in this kind of activity [73]. On the other hand, the implementation of a social chat between peers in treatments of smoking cessation has shown good results [48]. To increase users' motivation to help change their behavior, gamification elements were implemented [65].

For accomplishing the 5A's, the mHealth recommends breaking the habit and advices about the use of medication through recorded informative videos, podcasts, leaflets, and links to official websites. The plan for breaking the smoking habit is offered through the mHealth's dynamics by presenting the contents sequentially and informing the user of the plan objectives through both written information and the mHealth's website. Likewise, the programmed follow-ups were carried out through the feedback provided by the tool itself (informing them of the time they have been abstinent).

The techniques of the multicomponent CBT program were designed and operationalized through written information or audio recordings, short explanatory videos, and interactive learning activities (e.g., a recorded video of the person rejecting cigarettes). A space was also provided for taking notes (e.g., a list of reasons why I want to stay abstinent after hospitalization).

The implemented multicomponent program was distributed in 15 different boxes. Each box includes information about (1) contents, such as information about the components of tobacco, behavior analysis, or problem-solving techniques; (2) activities, such as relaxation, recording the pros and cons of quitting smoking, and social support; and (3) links with organizations or associations on smoking (Table 1).

**Figure 1:**
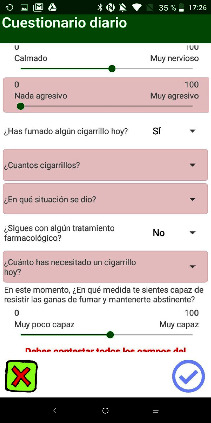
Screenshot of self-registration questionnaire the behavior analysis. The user answers the questions: Have you smoked any cigarettes today? In what situation did it occur? Are you still on any pharmacological treatment? How much have you needed a cigarette? At this moment, to what extent do you feel able to resist the urge to smoke and maintain abstinence?

**Figure 2:**
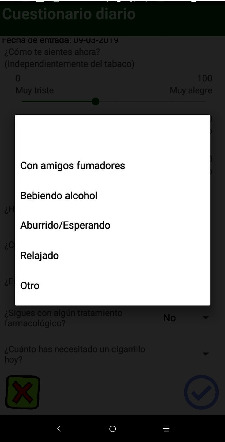
Screenshot of situations triggering behavior: “with friends who smoke”; “drunk alcohol”; “bored/waiting”; “relaxed”; and “other”.

The behavior analysis technique takes the form of a daily self-record, which is presented through a questionnaire to document the following (Figure 1): the number of cigarettes smoked, the situations that triggered consumption (Figure 2), the user's emotional state with regard to three emotions (anxiety, mood, and anger), the desire to smoke, the user's perceived self-efficacy in maintaining abstinence, and the monitoring of the pharmacological treatment.

**Figure 3:**
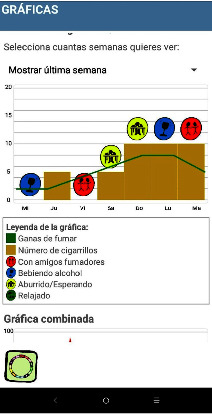
Screenshot of graphical resources describing smoking behavior analysis. In the columns of the graph you can see the “number of cigarettes smoked” (yellow square) and the green line indicates the “desire to smoke” (green square). The circle-shaped labels show the situations: red circle “with smoking friends”; blue circle “drinking alcohol”; yellow circle “bored/waiting”; and green circle “relaxed”.

All of these variables are measured on a sliding scale from 0 to 100 and they are represented in graphics that provide the user with feedback from the behavior analysis (Figure 3).

**Figure 4:**
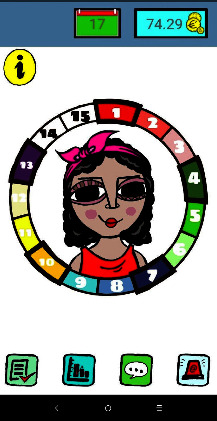
Screenshot of the main NoFumo+'s screen.

The mHealth starts with a main screen with a drawing of an avatar surrounded by a circle divided into 15 numbered boxes. At the end of the treatment the 15 boxes are colored (Figure 4).

**Figure 5:**
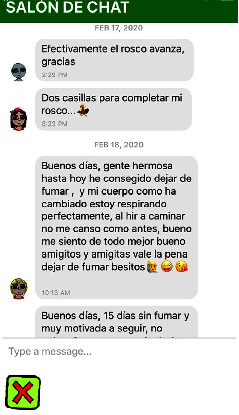
Screenshot of the social chat in the text, where 3 different mHealth users share their achievements; “Two boxes to complete my bagel…,” “Good morning people, until today I have managed to quit smoking…and my body has changed and I breathe perfectly…when I go for a walk I don't get tired like before I feel better all around, well my friends, it is worth quitting smoking.”

As the user progresses through the sessions daily, following a programmed 4-week sequence, 2 days for each box. Each box indicates a treatment session, and three boxes make up a phase. There are a total of five phases, and the change to the next phase can only be made after correctly answering a questionnaire that evaluates the contents of reviewed that phase. To encourage social support, an icon was designed to provide direct access to a “chat room” among the mHealth's users, available 24 hours a day, moderated by a psychology professional (Figure 5).

Among the motivational techniques of the program, personalized text messages are used, accompanied by a video “gift” represented by healthcare professionals who congratulate users for their abstinence or encourage them to return to abstinence if they have smoked. The motivational messages are all positive and they will appeal more to fear according to the response than to the user's assessment of perceived self-efficacy to remain abstinent (available in the self-registration). Likewise, to reinforce abstinence, the mHealth is complemented with basic elements such as a counting calculator (days without smoking, money saved, and cigarettes not smoked) and the possibility of sharing personal achievements through social networks.

In addition, users were able to make a call directly from the mHealth to their mobile contact list to a person who could provide support in critical situations.

The gamification included in the mHealth has the following two basic objectives [65]: for the user to complete the treatment offered by the mHealth and to reinforce the number of days of abstinence. To maintain adherence to the treatment, as a game element, an avatar (drawing of a bust) was designed that simulates the smoker.

Avatar's coloring improves weekly, appears on the screen in a greyish color at the beginning of the treatment, simulating the presence of carbon monoxide (CO) in the blood. At the end of the treatment, if abstinence is achieved, the avatar changes to a pink color that represents the elimination of CO acquiring a healthier appearance. The mHealth also offers rewards in the form of accessories to complement the avatar such as a mouth, hair, hat, and clothing. For “craving” situations, it offers the possibility of connecting to online games that foster distraction at those times.

### 2.2. mHealth's Technical Development

A multidisciplinary team was made up of professionals with training and experience in the area of healthcare, research, and technology with competencies to design a mHealth [74]. Specialized healthcare professionals from the Smoking Cessation Unit (SCU) (a psychologist, two nurses, and two pneumologists) considered the development of a digital tool to intervene in the treatment of smoking in hospitalized smokers. This team contacted researchers studying CBT for smoking cessation from the University of Balearic Islands (UIB). Subsequently, the Balearic Islands Technological Foundation (Bit Foundation), an external institution which has an agreement with the Balearic Public Health Service, was contacted. Bit Foundation provided two computer engineers for the mHealth development. The team met weekly to establish key concepts and constructions that should be included in the mHealth.

The healthcare professionals and the researcher from the UIB were responsible for agreeing on and adapting the contents of the standardized CBT program to the format of a Smartphone application. The UIB researcher proposed the design and dynamics of the treatment through the application (sequence and order of presentation of the contents). The pneumologists developed videos, podcasts, and informative texts about the way tobacco affects the lungs and the evidence-based pharmacological treatments. The nurses developed videos, podcasts, and leaflets about the components of tobacco and the health benefits of quitting smoking. In addition, they contacted other hospital specialists (cardiologist, nutritionist, and physiotherapist) to program videos and informative texts about tobacco's effect on the cardiovascular system, recommendations for weight control, physical training, and respiratory care. The psychologist proposed and developed the activities to be included in the app for each session (e.g., record of behavior analysis, writing down the reasons for deciding to stop smoking, record of alternative activities to smoking behavior, and management of social support through a chat). In addition, the psychologist contacted different experts in psychology to develop psychoeducation techniques for abstinence syndrome, problem-solving techniques, training in cigarette rejection skills, identification of negative thoughts, and cognitive restructuring.

In order to functionally adapt the experts' treatment proposal, the software engineers created the executive environment and framework for the mHealth development. Different Javascript tools we used for the server, such as Node.js runtime environment, and the framework for development of mobile applications Expo (which in turn is based on the React Native framework). The use of React Native (through Expo) allowed the same program code (written in Javascript) to generate the executables required by each operating system (Android or iOS). Once it was verified that the code worked with both operating systems, the executables for each system were generated using the pertinent native libraries. Regarding the design characteristics, icons with universal shape and color codes were used for different functions, such as information, graphics, chat, and emergencies. Once the executables were generated, the mHealth was uploaded to “Google Play” for the Android system, and to “App Store” for iOS. The mHealth was only available for smartphones with Android (version 5.0 onward) or Apple (version 4.0 onwards) operating systems. Finally, all the team members agreed on the user-application interaction variables to be collected, in order to carry out a subsequent study on the efficacy and mHealth efficiency [75]. Furthermore, the software engineers added the possibility for that, the research team to send direct notifications to the user's Smartphone through the interface (for example, reminders to perform the application's activities, such as the daily questionnaire, use of the chat, and propose greater help).

The mHealth was named NoFumo + [“I smoke no more”] and has been developed in compliance with the provisions of the Spanish Organic Law on Data Protection (IB3865), not only regarding the regulation of technical aspects of data protection but also the consent that must be requested for data process and analysis. Patients can access all the mHealth registered data at any time through a web-type platform created for this purpose.

### 2.3. Phase 1: Testing Phase

*Participants.* A panel composed of 15 psychologists, 13 of them women, was intentionally recruited, with a mean age of 26 years. All of them were studying Information and Communication Technology (ICT) in Health, belonging to the Master of General Health Psychology at the UIB. This group of psychologists was recognized as experts in the field due to their completion of a postgraduate degree, which included formal education in clinical psychology (CBT intervention). In addition, they received training in mHealth evaluation, particularly focused on smartphone apps.

*Assessment protocol.* The app assessment by the panel of experts had the objective of evaluating the presence or absence of quality and safety criteria a mHealth should have. As an evaluation protocol, the panel used the guidelines of the Andalusian Health Quality Agency (Spain) [50]; specifically, the adaptation made by Andújar-Espinosa et al. [76], which added criteria to evaluate gamification elements. The protocol consists of 31 dichotomous items (presence/absence) that are distributed in 7 categories: (1) the mHealth's functioning and the usability of its interface (1 item); (2) the relevance and suitability of its elements (1 item); (3) the quality of the contents (based on scientific evidence) and the safety of the information (whether it involves health risks, etc.) (3 items); (4) the services provided (technical support) (1 item); (5) the confidentiality and privacy (guarantee of anonymity and data treatment) (2 items); and (6) the gamification elements (rewards, avatars, badges, levels, challenges, etc.) (11 items). Given that the app was designed within the CBT framework, the research team added 6 items to the protocol for assessing the behavior modification techniques implemented.

The assessment protocol used shows a high degree of similarity with the Mobile App Rating Scale (MARS) [77] that includes the following dimensions: engagement, functionality, aesthetics, information, and subjective quality. In general, almost all MARS contents are subsumed in the protocol of this study. The inclusion in the mHealth of innovative elements such as gamification, not specifically contemplated by the MARS scale, led to the decision to opt for the Spanish protocol.

#### 2.3.1. Procedure

The computer engineers and software developers checked the overall operation of the mHealth before the evaluation of the panel of experts to avoid system errors and not delaying the process. The experts' panel assessment procedure took place on the university premises. In a 4-hour session, the healthcare professionals described the standardized multicomponent CBT program to quit smoking and the quality and safety criteria a healthcare app should have, according to the protocol. The participating experts downloaded the application onto their Smartphones and tested the mHealth without receiving any prior explanation. Thus, they had complete freedom to analyze its usability and interaction efficiency in the. Afterwards, the app's functioning was explained and doubts were resolved. The panel had a total of 30 days to test the application, fill out the checklist, and send, via email, comments or suggestions for improvement that were not originally included on the protocol.

### 2.4. Phase 2: Field Test Usability

#### 2.4.1. Participants

A group of smokers (*n* = 10, 4 men and 6 women) who had completed a group CBT smoking cessation treatment at a health center were recruited to participate voluntarily and without any economic incentive in the field study. They had just quit smoking. The mean age of the participants was 48.5 years (SD = 8), with an average consumption before the group treatment of 21 cigarettes per day. None of them reported having any previous experience with an mHealth.

#### 2.4.2. Assessment Protocol

The main objectives of the field study were to assess potential users' opinions about (1) the usability of the application, (2) the comprehension of the contents, and (3) the degree of satisfaction with the mHealth as a tool to quit smoking. To meet the first objective, a questionnaire adapted to users' understanding was designed based on the criteria recommended by the TIC Social Health Foundation of the Generalitat of Catalonia (Spain) [51]. This questionnaire is composed by 39 dichotomous items (yes/no) that measure usability and accessibility. It reasonably matches the scales of operation, ease of use, navigation, gestural design, and aesthetics (layout, graphics, and visual appeal) of the user version of the MARS scale (uMARS) [78]. To answer the second and third objectives, a telephone interview was carried out, consisting in two dichotomous questions (yes/no): (1) “Have you understood all the information and techniques that health professionals have given you through the videos?”, and (2) “Do you think that the app can be useful to help you quit smoking?” Also, possible comments to both questions were collected. Additional telephone interviews were conducted to gather supplementary data (available here) on understanding all the information and the perceived utility of the app in smoking cessation. During the field study, the research team also tested the operation of the application interface to assess whether the computing platform collected all user data correctly (questionnaires and notifications).

#### 2.4.3. Procedure

The research team contacted the coordinators of a Primary Care center that was conducting stop smoking group therapy. Once the objectives of the study had been explained, the center accepted and offered the possibility for a group of smokers to participate in the field study for the usability testing of the mHealth. After obtaining the consent of all the participants, they were individually contacted by phone to provide them with the codes to download the mHealth. Employing a mixed-method approach, once the results of the mHealth evaluation had been obtained, we collected additional qualitative information through telephone interviews, enabling the acquisition of more precise data than what was gathered through the questionnaires [79].

### 2.5. Design and Statistical Analysis

A quantitative descriptive design is suitable for this study, considering the various evaluations included in both phases of the mHealth development process. Basic descriptive statistics were used to manage the variables of scale. All analyzes were carried out using SPSS 25.0.

## 3. Results

This section presents the results of the two phases of the evaluation protocol.

### 3.1. Results of Phase 1: Testing with Experts

Seventy-three percent (11/15) of the experts recruited in phase 1 completed the NoFumo + mHealth evaluation using the assessment protocol.  Table 2 summarizes the obtained results in terms of agreement as well as the suggestions for the mHealth improvement.

**Figure 6:**
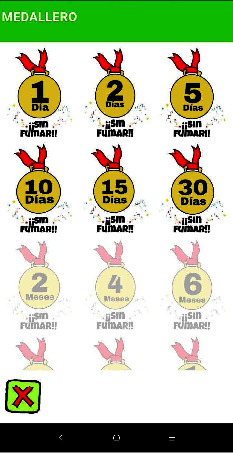
Screenshot of medal table shows medals earned from 1 day without smoking, 2 days without smoking…30 days without smoking.

As Table 2 shows, the majority of the experts easily located all the variables on the checklist and positively rated the application, describing it as being easy and intuitive to use. However, some suggestions for improvement should be highlighted, such as: (1) incorporating a tutorial video to complement the written explanation of the mHealth's functioning; (2) incorporating a notification indicating that its use is not a health risk; and (3) adding some other gamification insignia, such as a medallion, to reinforce long-term adherence, lasting up to five years, through a medallion (Figure 6).

### 3.2. Results of Phase 2: Usability Testing with Smokers

For the field study with the group of smokers who completed the treatment, valid results were obtained from 80% (8/10) of the smokers contacted. The evaluations obtained using the assessment protocol indicated that 100% (10/10) of the smokers identified a good quality level of the usability and accessibility criteria in the mHealth. Regarding the results of the telephone interviews, all of them (10/10) indicated that they were satisfied with the application and 83% (8/10) of the participants valued the comprehensibility of the contents positively. Also, all users (10/10) rated positively the information provided by the professionals through videos, highlighting that they are short and concise. We highlighted the following comments from participants: they pointed out the advantage of having the information and own behavior feedback available at all times through the mHealth; in regards of the app Google Fit, two participants mentioned that they used it and another participant said not to do it because he already had another mHealth. Finally, the effects of gamification elements were quite gratifying for users, in their opinion; these elements highly increased their motivation to use NoFumo + mHealth.

The research team tested the interface while participants were assessing the NoFumo + mHealth and confirmed that the platform correctly collected all the data (Table 3) and that the notifications functioned adequately. Table 3 collects the interface variables that show user interaction with the application, such as daily questionnaire, daily activity, connections to social chat, use of the emergency button, and evaluations on the use of the mHealth. It was also verified that 70% (7/10) of the participating smokers used the chat correctly. However, the team indicated that users' identification in the chat could be improved by adding their avatars as profile picture.

Throughout the field study, small functionality errors were also detected and corrected, such as difficulty downloading the mHealth due to lack of space on their personal smartphones, problems with entering the passwords to download NoFumo+, and excessive complexity of some of the evaluations to move through the treatment phases, which frustrated some users. Similarly, some content was modified or removed from the definitive mHealth; in the former case because it was not understood by the group of smokers and in the latter case because it was considered too extensive. As a final proposal for improvement, a “YouTube” type page was designed with tutorials on the activities and the mHealth's functioning.

## 4. Discussion

This paper presents the development of the mHealth “NoFumo+” to quit smoking that incorporates the main components of CBT and the recommendations of the CPG, as brief advice [8, 9]. The mHealth was developed by a multidisciplinary team and uses the potential of smartphones. Two pilot studies were carried out prior to its implementation. Finally, a clinical trial was conducted to validate the application in hospitalized patients, which results have been published [80].

There are other mHealths with the same purpose, such as Quit Genius, Smart Quit, or Smoke Mind, which also use CBT and CPG [81–83], although one of them (Smart Quit) does not use CBT as its main therapy, but instead uses acceptance and commitment therapy (ACT). For this reason, its intervention techniques are more focused on ACT, and publications on this mHealth do not clearly define how they perform the behavior analysis [82, 84], the main element of CBT. In the case of the other two applications (Quit Genius and Smoke Mind) each performs the behavior analysis differently. Quit Genius uses a questionnaire that records the variables and provides feedback on a graph. Smoke Mind uses a phone call from a professional, a specialist in CBT, who contacts users when they have smoked a cigarette. Online access to professionals is available in these two mHealths. Quit Genius facilitates communication with a “coach” the user can access during the treatment to reinforce motivation. NoFumo + starts with the record, as Quit Genius does, and increases its intensity in the intervention through the behavior analysis, according to its efficacy results. It also facilitates contact with a professional through a chat or by email.

It should be noted that, in the three applications mentioned above, users have to make an additional payment to access most of the contents and techniques (Smart Quit and Quit Genius) or have a private health system (Smoke Mind). However, NoFumo + offers this personalized support within a free public health system.

Another important difference between NoFumo+ and the three Apps mentioned above is the social support. These applications use social networks to share achievements as their only support. NoFumo + adds a chat between users moderated by a professional, thanks to the use of online applications that allow it. Another feature of our mHealth is that it uses the potential of smartphones to provide greater support or encourage effective activities, such as increasing physical activity. Perhaps it is these additional elements that result in better adherence rates on NoFumo+ (42%) [80] compared to other applications such as Smart Quit (24%) [84].

As a final result, NoFumo + maintains the elements of other mHealths, but we believe that it also tries to fill their gaps. This may be the reason why Nofumo + has achieved better abstinence rates (90.58%) 2 months after the end of treatment [80] than Smart Quit app (28%) [84].

The research team, recognizing the importance of evaluating the role of gamification in smoking cessation applications [85] and following expert recommendations, decided to incorporate additional gaming elements with a competitive dimension (e.g., a medal system and view their avatar's status in the chat). This approach is grounded in the premise that findings on mHealth with gamification factors may motivate both healthy and unhealthy individuals to maintain their health status [86]. It is worth noting that, although some research supports the effectiveness of these interventions, there is a scarcity of mobile applications for smoking cessation that incorporate gaming elements, and more clinical trials are needed to assess this function [85].

It is worth noting that the two pilot studies were carried out prior to its implementation, both pilot study with the experts' panel, and the study with smokers were very useful for improving the functionality of the mHealth and helping to solve arising problems.

### 4.1. Limitations

First, in regards the questionnaire about the mHealth testing, the choice of response options (yes/no) for the experts and users may have limited the variability in their responses. For future research, 5- or 7-point scale or qualitative methods could be used. Second, the field study was carried out with smokers who requested help in their health center because the mHealth has been designed within the framework of a public health program for smokers who suffer a hospital admission and, therefore, a period of forced abstinence. Smokers who do not seek help and lack sufficient self-motivation to face therapy should be recruited as well… Third, the sample size used in phase 2 is too small to extrapolate to the entire smoking population. However, the good results regarding the usability of the mHealth seem to indicate that it is easily usable by the smoking population with the same characteristics of the sample. Finally, it is important to note that the mHealth is currently only available in Spanish although it will be adapted to English.

The results of the clinical trial of the NoFumo + mHealth indicate that it can be an effective tool in the treatment of smoking cessation in the hospital setting. However, the global pandemic of COVID-19 limited the recruitment process, so it is necessary to conduct the study on larger samples, which will allow the analysis of the functions of the mHealth that show greater effectiveness.

## 5. Conclusions

After developing NoFumo+, an innovative quit smoking CBT-based mHealth including gamification elements, in the immediate future we plan to carry out a clinical application and evaluate its efficacy. As some authors point out [75, 87], efficacy studies have to be evidence-based and, thus, allow comparison with other mHealth Apps with homogeneous interventions. Although the first version of the NoFumo + mHealth was designed to be used with hospitalized patients, the research team will extend it to the general smoking population, depending on the results obtained. This mHealth could be adapted to be used in other contexts, not only hospitalized patients. This would require modifying the content (activities and examples) to the new given contexts, as well as the possibility of combining NoFumo+ with face-to-face visits if necessary. The final objective is to make this smartphone mHealth an efficient and affordable mobile tool to quit smoking and improve people's health.

## Figures and Tables

**Table 1:** Smartphone mHealth multicomponent program; behavior modification techniques.

Box	Information (text/audio)	Contents (short videos and leaflets)	Activities (interactive and notes)	Links (internet/web pages)
1	Treatment plan (establish objectives). Stimulus control (“golden rules”)	Tobacco components; nicotine; controlled breathing	Relaxation training; encouraging social support (friend/family); recording benefits for those who quit smoking now	Recommendations to stop smoking. https://www.altadis.com/tabaco-y-regulacion/fumar-y-salud/
2	The analysis of smoking behavior. Prevention of response. Advice about change of routine	Health effects of tobacco, pharmacological advice	Write down reasons to stop smoking, encourage social support	Information on how tobacco affects health: https://www.youtube.com/watch?v=ZAq0aNH7T88
3	Behavior analysis; the desire to smoke. Contents: activities	Abstinence syndrome. Advise on coping strategies	Select and/or note optional activities	Alternative coping strategies for withdrawal symptoms https://espanol.smokefree.gov/sintomas-de-abstinencia
4	Maximize the self-rewarding experience. Contents	Physical benefits of quitting smoking. Social support. Distraction. Prevention of response	Activities: Training in coping skills for risk situations	Benefits of quitting smoking. https://www.cdc.gov/tobacco/quit_smoking/how_to_quit/benefits/spanish/index.htm
5	Analyze smoking behavior and desire to smoke, personal reasons to quit. Feedback graphics	Identify risk situations	Relaxation (jacobson and autogenous)	Controlled breathing, self-guided relaxation technique (jacobson). https://m.youtube.com/watch?v=ZwOl80h_jEA
6	Remember to control stimuli that remind you to smoke (e.g., situations, smoke-free spaces …)	Effective pharmacological treatments to stop smoking	Activities to maintain abstinence	Life without tobacco. https://m.youtube.com/watch?v=8Vom45iDUXQ
7	History of tobacco. Raising awareness in tobacco advertising. Assert yourself as a nonsmoker	Informing about the history of tobacco, psychological part of addiction	Alternative activities for risk situations; social support	Harmful advertising about smoking. https://m.youtube.com/watch?v=Gf6Kbje_nTI
8	Time management. Graphical feedback of behavior analysis	Social support (chat). Distraction	Smoking behavior analysis charts	Common myths. https://pnsd.sanidad.gob.es/ciudadanos/informacion/tabaco/menuTabaco/mitosRealidades.htm
9	Negative thoughts	Advice about errors in thinking and their modification; social chat	Write down negative thoughts and modify them; talk to yourself	Change in thinking
10	Stress management	The problem-solving technique	Technical problem-solving training	Problem-solving technique. https://m.youtube.com/watch?v=OXt2XTAPrr0
11	Advise about social skills as a technique	Talking to oneself: Thought-stopping technique; social skills for cigarette refusal	Training in social skills	
12	Reinforcing abstinence as a reward and objective of the program	Cognitive restructuring: true story OD chronic obstructive pulmonary disease (COPD)	Action in the event of a fall or/and relapse Testimony one ex-smoking COPD (5 years abstinence)	The reason for the fall and relapse https://m.youtube.com/watch?v=M28lUeqzDeY
13	Strengthen the ex-smoker's identity (smoking is not an option)	The urge to smoke, the temptation	Testimony of 2 ex-smoking patients (1 year and 25 years of abstinence)	Resources/testimonies from other ex-smokers. https://m.youtube.com/watch?v=M28lUeqzDeY
14	Reinforcing alternative smoking behaviors	Physical exercise of different intensities. Psychoeducation and behavioral activation	Use a pedometer	Program to promote physical exercise at different ages. https://www.estilosdevidasaludable.mscbs.gob.es/actividadFisica/actividad/daElPaso/comoLoHacen/home.htm
15	Reinforcement and reobjectives of the treatment in terms of monitoring in person and through the application	Weight control. Training in planning (diet and exercise)	Harvard plate	Healthy eating education and recipes https://www.hsph.harvard.edu/nutritionsource/healthy-eating-plate/translations/spanish/

**Table 2:** Results and suggestions about NoFumo + mHealth from the experts' panel according to the assessment protocol (*n* = 11).

Variables^a^	Indicators^b^	Results *n* (%)^c^	Suggestions^d^
Functioning	Easy to use	11 (100%)	Change the format to a video tutorial or web page

Relevance	Purpose and clarity in the objectives pursued	11 (100%)	

Quality and security of information	Appropriate to the audience	11 (100%)	Add the possibility of being able to listen to the information, as well as read it
	Transparency about owners	11 (100%)	
	Reliability contained	6 (54%)	Add the sentence: “The app is not harmful to health”

Providing services	Health risk they may have with the use of the application	11 (100%)	Add a video on how to use the app

Confidentiality and privacy	Technical support and assistance on its handling	8 (72%)	Unify all information dealing with this indicator
	Privacy/data protection	11 (100%)	

Gamification elements	Data processing	11 (100%)	
	Rewards	11 (100%)	
	Awards	11 (100%)	
	Avatars	8 (72%)	Add a contest, create competition
	Classification tables	10 (90%)	
	Levels	6 (54%)	
	Challenges	7 (63%)	Add medallion
	Points	11 (100%)	
	Feedback	10 (90%)	
	Goals	11 (100%)	
	Social interaction	11 (100%)	

Behavior modification techniques	Achievements	11 (100%)	
	Feedback	11 (100%)	
	Self-monitoring	11 (100%)	
	Behavior comparison	11 (100%)	
	Rewards and threats	11 (100%)	
	Incentive	11 (100%)	

BCT techniques	Self-observation: Self-registration and graphic representation of consumption	11 (100%)	
	Information on tobacco	11 (100%)	Add attractive text format, leaflets
	Stimulus control	11 (100%)	
	Activities to avoid nicotine withdrawal symptoms	8 (72%)	
	Physiological feedback from cigarette smoking	4 (36%)	Add other indicators (e.g., co-oximetry)
	Social commitment	11 (100%)	
	Relapse prevention strategies	11 (100%)	

^a^Variable to be measured. ^b^The indicators of each variable. ^c^Number of psychologist who locate the variable (*n*); sample the percentage of psychologists who locate the variable (%). ^d^Suggestions for improvements by users.

**Table 3:** Elements and variables included in the mHealth's interface.

Elements	Variables
Diary questionnaire	(i) Number of times the user enters data
	(ii) Emotions 0–100; calm-nervous/cheerful-sad/nonaggressive
	(iii) Smoking: yes/no
	(1) Number of cigarettes (number)
	(2) Situations (other smokers, drinking alcohol, bored, relaxed, other)
	(iv) Pharmacological treatment: yes/no
	(1) Treatment (patches, varenicline, other)

Activity	(i) Number of boxes fulfilled
	(ii) Duration of the activity (min): from when it is switched on to when it is switched off
	(iii) PDF read: if you open it (yes/no)
	(iv) Video display time: if you do not stop it, it continues to count
	(v) Additional material link: (yes/no)

Chat connections	(i) Date
	(ii) Time spent in the chat

Emergency button	(i) Date
	(ii) Length of stay
	(iii) Telephone call
	(iv) Online games
	(v) Send email to the service yes/no

Evaluation/gamification elements	(i) If user passes (evaluation: 1/2/3/4/5): yes/no (obtains gamification)
	(ii) Number of attempts to pass the evaluation

## Data Availability

The data used to support the findings of this study are available on request from the corresponding author.
