# Supplementary material for: NoFumo+: Mobile Health App to Quit Smoking Using Cognitive-Behavioral Therapy
Source: Nurs Res Pract. 2024 Sep 26;2024:8836672. doi: 10.1155/2024/8836672 (PMC11449556; doi:10.1155/2024/8836672)
Supplement: Supplementary Materials — Supplementary material is available for this article online (S1). [file 8836672.f1.pdf]

NoFumo+

- ✓ 15 boxes/ 30 days
- ✓ 3 boxes/ 1 phase

Sessions

- ✓ Psychoeducational (video, triptych, audio)
- ✓ Activities
- ✓ Additional information (links)

Week 1 | Week 4

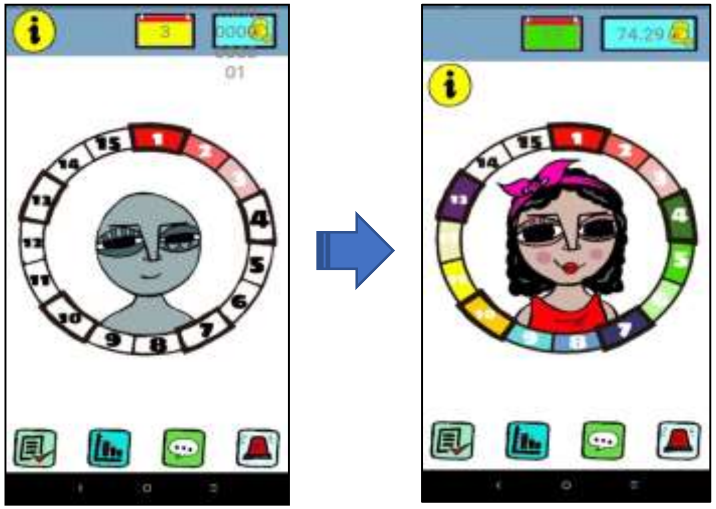

Emergency

- ✓ Remember coping strategies (video)
- ✓ Call the support contact
- ✓ Send email to health personnel
- ✓ Distraction online games

Additional activities

- ✓ Participate in social support chat
- ✓ To share achievements by social networks
- ✓ Record physical activity (steps counter)

Self-registration (daily)

- ✓ Emotions. How you feel?
- ✓ Cigarretter consumption
- ✓ Craving
- ✓ Self-efficacy expectations

Self-evaluation (weekly)

- ✓ 3 questions with 3 response alternatives

→ Progress charts
→ Motivational messages
→ Encouragent GIFs

→ Phase change
→ Avatar enhancement

→ Review information from previous sessions
